# A World Health Organization tool for assessing research ethics oversight systems

**DOI:** 10.2471/BLT.24.292219

**Published:** 2025-05-03

**Authors:** Carl H Coleman, Alireza Khadem, John C Reeder, Hiiti B Sillo, Rogerio Gaspar, Andreas Reis

**Affiliations:** a Seton Hall Law School, 1109 Raymond Blvd, Newark, New Jersey, 07102, United States of America.; b Access to Medicines and Health Products, World Health Organization, Geneva, Switzerland.; c Research for Health, World Health Organization, Geneva, Switzerland.

## Abstract

Although most countries have ethical oversight systems for health-related research involving human participants, mechanisms for assessing the quality of those systems are not regularly used, particularly in low-resource settings. To address this gap, the Regulatory System Strengthening, Regulation and Safety unit and Health Ethics and Governance unit of the World Health Organization (WHO) recently released a tool for benchmarking ethics oversight of health-related research involving human participants. The tool provides a simple, easy-to-measure set of indicators for assessing the quality of research ethics oversight systems without the need to invest a great deal of resources. The tool comprises 48 indicators divided across three areas: (i) the national context; (ii) research ethics committees; and (iii) institutions that conduct health-related research involving humans, such as academic medical centres. Indicators related to the national context are intended to be evaluated in a single assessment applicable to the country as a whole, whereas indicators related to research ethics committees and research institutions are evaluated on an entity-by-entity basis. Some countries may choose to assess a representative sample of research ethics committees and institutions; alternatively, national authorities might ask research ethics committees and institutions to undertake self-assessments and report the results. Research ethics committees or institutions could also use WHO’s tool on their own as part of a process of quality improvement. WHO is working with global partners to disseminate the tool and support global implementation. Widespread use of the tool is expected to enhance policy coherence in ethics oversight and facilitate multinational research.

## Résumé

La plupart des pays disposent de systèmes de supervision de l’éthique dans le domaine de la recherche scientifique en lien avec la santé et impliquant des participants humains. Toutefois, les mécanismes d’évaluation de la qualité de ces systèmes ne sont pas mis en pratique sur une base régulière, en particulier dans les pays à faibles ressources. Pour combler cette lacune, l’unité Renforcement des cadres réglementaires, réglementation et sécurité ainsi que l’unité Éthique et gouvernance de la santé de l’Organisation mondiale de la santé (OMS) ont récemment publié un outil d’évaluation comparative de la surveillance de l’éthique en matière de recherche dans la santé impliquant des participants humains. Cet outil fournit un ensemble d’indicateurs simples et faciles à mesurer pour évaluer la qualité des systèmes de surveillance de l’éthique dans le domaine de la recherche sans avoir à investir une quantité importante de ressources. Il comprend 48 indicateurs couvrant trois domaines: (i) le contexte national; (ii) les comités d’éthique en matière de recherche; et (iii) les institutions menant des recherches dans le domaine de la santé impliquant des êtres humains, telles que les centres médicaux universitaires. Les indicateurs relatifs au contexte national sont destinés à être évalués dans le cadre d’une analyse unique applicable à l’ensemble d’un pays, tandis que les indicateurs relatifs aux comités d’éthique en matière de recherche et aux institutions de recherche sont évalués entité par entité. Certains pays peuvent choisir d’évaluer un échantillon représentatif de comités d’éthique et d’institutions de recherche; les autorités nationales peuvent également demander aux comités d’éthique et institutions de recherche de procéder à des auto-évaluations et d’en communiquer les résultats. Les comités d’éthique ou institutions de recherche peuvent également utiliser l’outil de l’OMS dans le cadre d’un processus d’amélioration de la qualité. L’OMS collabore avec des partenaires mondiaux afin de diffuser cet outil et de soutenir sa mise en œuvre à l’échelle mondiale. L’utilisation généralisée de l’outil devrait renforcer la cohérence des politiques en matière de surveillance de l’éthique et faciliter la recherche multinationale.

## Resumen

Aunque la mayoría de los países dispone de sistemas de supervisión ética para la investigación en salud que involucra a participantes humanos, los mecanismos para evaluar la calidad de estos sistemas no se utilizan de manera regular, en particular en contextos con recursos limitados. Para abordar esta carencia, la Unidad de Fortalecimiento de los Sistemas Regulatorios, Regulación y Seguridad y la Unidad de Ética y Gobernanza en Salud de la Organización Mundial de la Salud (OMS) han desarrollado recientemente una herramienta para la evaluación comparativa de la supervisión ética de la investigación en salud con participación humana. La herramienta proporciona un conjunto sencillo y de fácil medición de indicadores para evaluar la calidad de los sistemas de supervisión ética de la investigación, sin requerir una gran inversión de recursos. La herramienta consta de 48 indicadores distribuidos en tres áreas: (I) el contexto nacional; (ii) los comités de ética en investigación; y (iii) las instituciones que realizan investigaciones relacionadas con la salud en seres humanos, como los centros médicos y académicos. Los indicadores relacionados con el contexto nacional están diseñados para ser evaluados en una única valoración aplicable a todo el país, mientras que los indicadores relativos a los comités de ética en investigación y a las instituciones de investigación deben evaluarse de manera individualizada. Algunos países pueden optar por evaluar una muestra representativa de comités de ética en investigación y de instituciones; como alternativa, las autoridades nacionales podrían solicitar que los comités de ética y las instituciones realicen autoevaluaciones y reporten los resultados. Los propios comités de ética en investigación o las instituciones también podrían utilizar la herramienta de la OMS de manera independiente como parte de un proceso de mejora de la calidad. La OMS está colaborando con socios globales para difundir la herramienta y apoyar su implementación a nivel mundial. Se espera que el uso generalizado de la herramienta contribuya a mejorar la coherencia de las políticas en materia de supervisión ética y facilite la realización de investigaciones multinacionales.

## ملخص

على الرغم من أن معظم الدول لديها أنظمة للرقابة على الأخلاقيات، للأبحاث المتعلقة بالصحة التي تشمل مشاركين من البشر، إلا أن آليات تقييم الجودة في هذه الأنظمة لا يتم استخدامها بانتظام، وخاصة في البيئات ذات الموارد المنخفضة. ولسد هذه الفجوة، فإن كل من وحدة دعم وتنظيم وسلامة النظام اللوائحي، ووحدة أخلاقيات الصحة والحوكمة، التابعتين لمنظمة الصحة العالمية (WHO)، قد أصدرتا مؤخرًا أداةً لمعايرة الرقابة الأخلاقية على الأبحاث المتعلقة بالصحة، والتي تشمل مشاركين من البشر. توفر الأداة مجموعة من المؤشرات البسيطة سهلة القياس، لتقييم جودة أنظمة الرقابة على أخلاقيات البحث دون الحاجة إلى استثمار قدر كبير من الموارد. تتكون الأداة من 48 مؤشرًا مقسمة على ثلاثة مجالات: (1) السياق الوطني؛ و(2) لجان أخلاقيات البحث؛ و(3) المؤسسات التي تُجري أبحاثًا متعلقة بالصحة وتركز على المشاركين من البشر، مثل المراكز الطبية الأكاديمية. من المقرر تقييم المؤشرات المتعلقة بالسياق الوطني في تقييم وحيد ينطبق على الدولة ككل، بينما يتم تقييم المؤشرات المتعلقة بلجان أخلاقيات البحث ومؤسسات البحث على أساس كل كيان على حدة. قد تختار بعض الدول تقييم عينة تمثيلية من لجان ومؤسسات أخلاقيات البحث؛ أو بدلاً من ذلك، قد تطلب السلطات الوطنية من لجان ومؤسسات أخلاقيات البحث إجراء تقييمات ذاتية وتقديم نتائجها. كما يمكن للجان أو مؤسسات أخلاقيات البحث استخدام أداة منظمة الصحة العالمية بشكل مستقل كجزء من عملية تحسين الجودة. وتعمل منظمة الصحة العالمية مع شركاء عالميين من أجل نشر هذه الأداة ودعم تطبيقها على مستوى العالم. ومن المتوقع أن يؤدي الاستخدام الواسع لهذه الأداة إلى اتساق السياسات في مجال الرقابة على الأخلاقيات، وتسهيل إجراء الأبحاث متعددة الجنسيات.

## 摘要

尽管大多数国家都针对涉及人类参与者的健康相关研究构建了伦理监督系统，但用于评估此类系统质量的机制尚未得以经常应用，特别是在资源贫乏地区。为了弥补这一缺陷，世界卫生组织 (WHO) 的监管体系加强、监管与安全部门以及健康伦理与治理部门最近发布了一种评估工具，用于对涉及人类参与者的健康相关研究的伦理监督工作进行基准评估。该工具提供了一套简单且易于衡量的指标，可用于评估研究伦理监督系统的质量，且无需投入大量资源。该工具包含涉及以下三大领域的 48 个指标：(1) 国家背景；(2) 研究伦理委员会；以及 (3) 开展涉及人类的健康相关研究的机构，例如学术医学中心。与国家背景有关的指标旨在用于对整个国家进行统一评估，而与研究伦理委员会以及研究机构有关的指标则用于对各个实体进行逐一评估。有些国家可能会选择对具有代表性的研究伦理委员会和机构进行评估；或者，国家主管当局可能会要求研究伦理委员会和机构进行自我评估并汇报评估结果。研究伦理委员会或机构还可独立使用 WHO 的工具，这是质量改进流程的一部分。WHO 正与全球伙伴开展合作以推广该工具并支持在全球范围内的使用。据预计，该工具的广泛使用有助于加强伦理监督方面的政策一致性并推动跨国研究。

## Резюме

Хотя в большинстве стран действуют системы этического надзора в сфере медицинских исследований с участием людей, механизмы оценки качества таких систем не имеют регулярного применения, в частности в странах с низким уровнем дохода. Для устранения этого пробела Всемирная организация здравоохранения (ВОЗ) недавно разработала инструмент для сравнительного анализа систем этического надзора в сфере медицинских исследований с участием людей. Данная инициатива была реализована через подразделение по укреплению систем нормативно-правового регулирования, нормативной базы и безопасности в сотрудничестве с подразделением по этике здравоохранения и методов руководства. Инструмент предоставляет простой и легкий в измерении набор индикаторов для оценки качества систем этического надзора в сфере исследований и не требует вложения значительных ресурсов. В него вошли 48 индикаторов, сгруппированных по трем областям: (i) национальный контекст, (ii) комитеты по этике исследований и (iii) учреждения, в которых проводятся медицинские исследования с участием людей (например, учебные медицинские центры). Индикаторы, относящиеся к национальному контексту, подразумевают единую оценку на уровне страны в целом, тогда как индикаторы, относящиеся к комитетам по этике и исследовательским учреждениям, применяются отдельно к каждому объекту оценивания. В некоторых странах предпочтение отдают формированию репрезентативной выборки исследовательских центров и комитетов по этике исследований, тогда как в иных случаях национальные органы власти могут попросить учреждения и комитеты по этике провести самостоятельную оценку с последующим предоставлением отчетности. Исследовательские учреждения и комитеты по этике исследований также могут использовать предложенный ВОЗ инструмент для проведения оценивания по собственной инициативе в рамках процесса улучшения качества. ВОЗ работает с международными партнерами с целью распространения этого инструмента и содействия в его внедрении. Ожидается, что широкое применение инструмента позволит согласовать политики в области этического надзора и упростит проведение многонациональных исследований.

## Introduction

A core requirement of international guidelines on the ethics of health-related research involving human participants is that an effective system of ethical oversight exists at institutional and national levels. Such a system should ensure that: (i) the proposed research is reviewed in advance by a competent research ethics committee; (ii) the research is monitored to verify that ethical requirements are being followed; and (iii) mechanisms are in place to promote accountability, transparency and public engagement. By now, most countries have established some sort of research ethics system. A few have developed mechanisms for overseeing the quality of those systems, including national bodies like the National Health Research Ethics Council in South Africa and regional collaborations like the SIDCER-FERCAP Foundation, which was established to promote the development of human research ethics.^1^^,^^2^ Yet, in most of the world, particularly in low-resource settings, mechanisms for assessing the quality of research ethics systems are not regularly used. 

To address this gap, in 2023, the Regulatory System Strengthening, Regulation and Safety unit and the Health Ethics and Governance unit of the World Health Organization (WHO) released a tool for benchmarking ethics oversight of health-related research involving human participants.^3^ This tool was developed under the guidance of an international expert advisory group, which included representatives of global networks of research ethics committees as well as leading researchers, regulators and academics from across WHO regions. A draft of the tool was first published on WHO’s website for comments and was subsequently revised to reflect the many insightful replies received. Before the tool was released, it was piloted and refined at workshops in Egypt, India, Kenya, Nepal, Nigeria and Pakistan, which together included participants from more than 100 research ethics committees, national ethics committees, national regulatory agencies and research institutions.

## Goals of the research ethics oversight tool

The primary purpose of the research ethics oversight tool is to provide a relatively simple, easy-to-measure set of indicators that can be used to provide a basic assessment of the quality of research ethics oversight systems in countries and institutions without the need to invest a great deal of resources. The tool is not a normative ethical document. Instead, it takes as a given the ethical commitments articulated in existing international guidance documents, including long-standing WHO guidelines for research ethics committees;^4^ the comprehensive set of ethical guidelines for health-related research involving humans jointly issued by the Council for International Organizations of Medical Sciences in collaboration with WHO;^5^ and two widely accepted declarations on research ethics issued by the World Medical Association.^6^^,^^7^ In essence, the tool is intended to provide a means of determining whether these guidelines are being adhered to.

The tool offers several potential benefits for health research and ultimately public health. First, the tool provides a basis for identifying areas in which a research ethics oversight system does not fully conform to internationally accepted ethical standards. Second, the tool makes it possible to establish benchmarks against which future improvements in system performance can be measured and to evaluate the impact of future policy changes. Third, the tool could potentially be used by partners in multinational research as a basis for confidently deferring to another country’s ethics review process, thereby reducing the number of unnecessary and time-consuming duplicative reviews. Finally, the tool provides a means for funders and others engaged in efforts to strengthen countries’ research ethics capacities to identify where resources could be most usefully used and to monitor the impact of any interventions they support.

One of the most important characteristics of a good set of indicators is that “the gathering of the required information must be technologically feasible and affordable and must not overburden the system.”^8^ With that goal in mind, rather than attempting to define a comprehensive set of quality measures, the tool focuses on a few basic indicators of quality that any well-functioning research ethics oversight system can reasonably be expected to possess. In developing the tool, an effort was made to strike a balance between the three major types of indicator: (i) those that assess the organizational characteristics of the system (structural indicators); (ii) those that assess the manner in which the system carries out its activities ( procedural indicators); and (iii) those that assess the extent to which the system achieves its objectives (outcomes-based indicators).

The research ethics oversight tool was modelled on WHO’s Global Benchmarking Tool,^9^ the primary means by which WHO evaluates and supports the strengthening of regulatory systems for medical products. The Global Benchmarking Tool consists of a series of indicators and subindicators related to the overarching national regulatory system and eight core regulatory functions: (i) registration and marketing authorization; (ii) vigilance; (iii) market surveillance and control; (iv) licensing establishments; (v) regulatory inspection; (vi) laboratory testing; (vii) clinical trials oversight; and (viii) national regulatory authority lot release. Together, these indicators are designed to cover the full life cycle of medical products.

More generally, WHO has long relied on indicators to measure adherence to internationally accepted ethical and regulatory standards. For example, WHO has developed indicators to monitor countries’ compliance with their obligations under the International Health Regulations.^10^ These indicators measure, on a scale of 1 to 3, the level of implementation of mechanisms for identifying and responding to global public health threats.^11^ Similarly, WHO has established indicators for assessing countries’ progress towards meeting the goals of the Framework Convention on Tobacco Control.^12^ Examples include indicators of: (i) whether countries have established and are enforcing smoke-free policies; and (ii) the extent to which countries have reduced morbidity and mortality from exposure to second-hand smoke.^13^

## Overview of the research ethics oversight tool

**Fig. 1:**
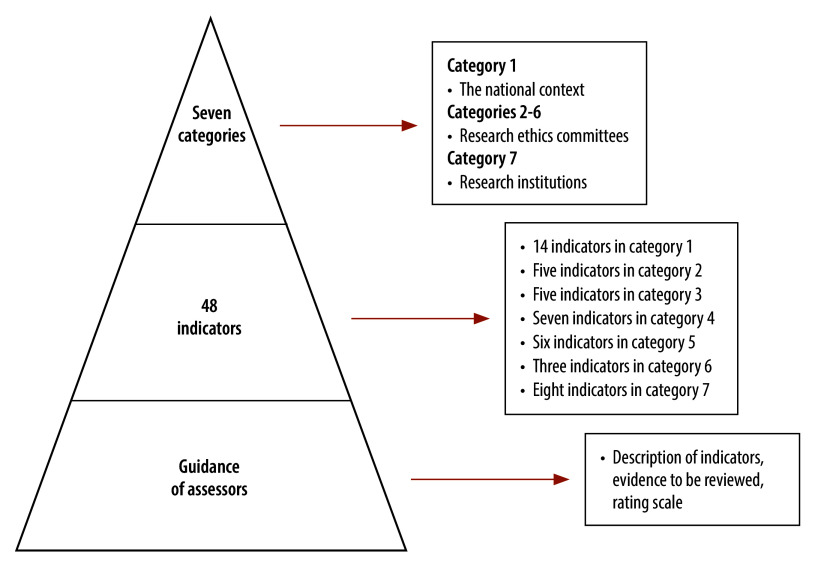
Structure of the WHO global benchmarking tool for strengthening research ethics oversight systems, 2023

The tool comprises seven categories and is designed to evaluate three aspects of the research ethics oversight system: (i) the national context; (ii) research ethics committees; and (iii) research institutions, which are defined as institutions, such as academic medical centres, whose employees or agents conduct health-related research with humans (Box 1). The seven categories cover the essential components of an effective research ethics oversight system, without which the system cannot be considered adequate. The international expert advisory group identified potential quality measures for each category through a brainstorming process, and ultimately settled on 48 unique indicators. For each of the 48 indicators, the research ethics oversight tool provides guidance for assessors, which includes a description of each indicator, examples of relevant evidence to review and a rating scale (Fig. 1).

Box 1 **Assessment categories in the WHO global benchmarking tool for strengthening research ethics oversight systems, 2023**

1. Legal provisions and regulatory framework
2. Structure and composition of the research ethics committee
3. Research ethics committee's resources
4. Research ethics committee's procedures
5. Mechanisms for promoting the transparency of the research ethics committee
6. Mechanisms for the research ethics committee to monitor its own performance
7. Research institutions

WHO: World Health Organization.

WHO: World Health Organization.

Note: The research ethics oversight tool’s categories are described in Box 1.

Indicators related to the national context are intended to be evaluated in a single assessment that is applicable to the country as a whole. Countries may choose to entrust these assessments to national authorities, such as a health ministry, a research ministry or another regulatory agency; or they could rely on external experts, for example, by commissioning a report from an academic institution. By contrast, indicators related to research ethics committees and research institutions must be evaluated on an entity-by-entity basis. In countries with a relatively small number of research ethics committees and research institutions, a single entity could feasibly conduct all the necessary assessments. However, in countries with an extensive research infrastructure and many local research ethics committees, the number of assessments required could reach into the hundreds. In these settings, national oversight authorities might assess a representative sample of research ethics committees and research institutions. Alternatively, national authorities might ask research ethics committees to undertake their own self-assessments and report the results. Research ethics committees might also choose to undertake self-assessments on their own initiative as part of a process of quality improvement. In general, externally conducted assessments are preferable because they help ensure that standards are applied consistently. However, when resources are constrained, self-assessments can be a cost-effective means of generating a large number of assessments over a relatively short time period.

## Assessing the national context

The indicators used to assess the effect of the national context on the quality of the research ethics oversight system are included in Category 1: Legal provisions and regulatory framework. Most of these indicators relate to the extent to which the basic elements of the research ethics oversight system have been codified into legally binding instruments, such as statutes, regulations, decrees or legally binding guidance documents. For example, one indicator relates to the existence of legal provisions that explicitly require health-related research involving humans to be reviewed and approved by a research ethics committee, with the possible exception of specified categories of low-risk studies. Reviewers are asked to examine a country’s laws to determine whether this requirement has been either: (i) fully implemented (that is, there exist legal provisions requiring a research ethics committee to review all health-related research involving humans before recruitment begins, with the possible exception of specified categories of low-risk research); (ii) partially implemented (that is, there exist legal provisions requiring a research ethics committee to review only some types of research, such as government-funded studies); or (iii) not implemented (that is, there are no legal provisions requiring research ethics committee review).

Some of the national indicators are designed to ensure that research ethics committees have adequate legal authority to conduct their mission. For example, one indicator relates to the existence of legal provisions that explicitly give research ethics committees the authority to suspend or terminate a study if a committee determines that the study no longer meets the criteria that justified its initial approval. Another relates to the existence of legal provisions which ensure that a research ethics committee’s decision not to approve a study cannot be overruled, except when a regulatory agency or court has determined that an abuse of authority has occurred.

The national indicators also ask whether a country has legal provisions ensuring that research participants have access to medical treatment for any injuries directly resulting from their participation in the research; and that participants and their dependents are protected from the financial consequences of any injury or death suffered by the participant as a direct result of their participation. This particular indicator is grounded in a broad international consensus, as reflected in influential documents such as the Declaration of Helsinki,^6^ and in the legal requirements of some jurisdictions (for example, the European Union).^14^ However, several countries have not yet fully implemented these provisions, including some that host many research sponsors.^15^

In addition to legal provisions, one indicator for the national context relates to the existence of national, subnational, multinational or local oversight authorities, or their combination, that support research ethics committees and ensure they adhere to applicable ethical and legal requirements. Depending on the country, the oversight authority might be a health ministry, another government agency or an independent agency created specifically to provide oversight of research ethics committees. For a country to be considered fully compliant with this indicator, not only must an oversight entity exist but there must also be evidence that the entity provides ongoing technical assistance, coordination and monitoring of research ethics committees.

## Assessing research ethics committees

The indicators used to assess research ethics committees are divided into five categories: (i) structure and composition; (ii) resources; (iii) procedures; (iv) mechanisms to promote transparency; and (v) mechanisms to monitor committees' own performance. Indicators related to structure and composition are designed to reinforce basic standards for the membership of research ethics committees, such as the necessary inclusion of nonscientific members, the appointment of members for a fixed term rather than indefinitely, and arrangements to ensure that members are not removed before their term expires unless they have been found to have substantially breached their duties. The research ethics oversight tool directs assessors to focus not only on what is stated in research ethics committee policies but also on the extent to which those policies are followed. For example, assessors are asked to determine whether the research ethics committee has a policy to invite nonmembers to contribute to the review of research that raises issues beyond the scope of the members’ expertise or experience, and also to determine how frequently nonmembers are actually invited to participate.

The indicators related to resources focus on: (i) whether the research ethics committee has sufficient and competent staff; (ii) whether members of the committee and staff receive initial and continuing training; and (iii) whether the committee has adequate facilities, equipment, technological support and financial resources. The research ethics oversight tool suggests the kind of evidence that can help assessors make judgments about the adequacy of resources. For example, with regard to financial resources, relevant evidence includes: (i) the sources of funding for the research ethics committee; (ii) the committee’s budget for the current and preceding year, with information on how those budgets were determined; (iii) information about any budget proposals by the committee that were denied, the basis for the denials and the impact of the denials on the committee’s operations; (iv) the number of applications reviewed annually by the committee; (v) committee member and staff surveys on the adequacy of the committee’s budget, if available; (vi) the committee’s annual financial report; and (vii) information about any activities that were not performed because of budgetary constraints. Ultimately, the assessor’s goal is to determine whether the research ethics committee has “adequate financial resources and reliable source(e)s of funding” and that the funding mechanism “does not create financial incentives to approve or reject particular studies.”^3^

The indicators related to procedures look at basic aspects of how research ethics committees conduct their activities. For example, assessors are asked to determine: (i) whether the committee has appropriate guidelines for the submission and screening of applications; (ii) whether it has written procedures to ensure that its deliberations adhere to relevant ethical criteria; (iii) whether it has adequate time before and during committee meetings for the meaningful review of research procedures; and (iv) whether it has procedures to ensure that decisions are made in a timely manner and are promptly communicated to the principal investigators. Reflecting WHO’s increasing emphasis on the need to conduct research rapidly during public health emergencies,^16^ assessors are also asked to determine whether the research ethics committee has procedures for ensuring the fast-track review of proposals in emergency situations.

Indicators in the areas of transparency and monitoring relate to specific types of information that research ethics committees should make publicly available (for example, committee guidelines and details of procedures and funding sources) or available on request (for example, a list of committee members and their titles, a list of principal investigators, and the dates on which research proposals were approved by the committee). Two indicators in these categories require assessors to determine the committee’s receptivity to questions and feedback from research participants and investigators. Relevant evidence for these indicators includes: (i) the availability of contact information for the committee on the committee’s website and on committee-approved consent forms; (ii) the existence of a comprehensive frequently-asked-questions webpage; and (iii) examples of actions taken by the committee in response to the feedback it has received.

Finally, the research ethics oversight tool includes three indicators that assess the extent to which research ethics committees proactively engage in efforts to monitor their own performance. The first indicator relates to the existence of: (i) mechanisms for obtaining feedback from investigators and research participants about their experience of the research studies; and (ii) mechanisms for instituting appropriate corrective actions in cases where problems are revealed. The second indicator relates to the existence of a system for ensuring the consistent and coherent application of policies and procedures. Such a system might include mechanisms such as regular debriefings with committee members and staff, or retrospective reviews of selected applications. The third indicator is more open-ended than the others; it relates to whether the research ethics committee regularly conducts internal reviews of its performance.^3^ The tool includes suggestions for ways in which a research ethics committee might engage in this kind of internal self-assessment but it does not recommend any particular mechanism. The point is simply to encourage committees to develop a practice of engaging in some form of self-critical analysis.

## Assessing research institutions

The institutional context in which research ethics committees do their work is crucial for their proper functioning. The category for research institutions is the shortest section of the research ethics oversight tool. The category is not designed to provide a basis for conducting a comprehensive assessment of institutional quality, but instead to identify a few markers of institutions’ commitment to protecting research participants. The Council for International Organizations of Medical Sciences has just released comprehensive guidelines on good governance practice for research institutions,^17^ which will be complementary to WHO’s tool.

Several of the indicators in this category seek to ensure that institutions provide adequate oversight of their affiliated researchers. For example, the tool assesses whether an institution verifies that all proposals for health-related research involving humans are submitted to a registered research ethics committee if any part of the research is to be conducted by a researcher affiliated with the institution. Other indicators relate to the existence of ethics training programmes for researchers, and of policies on researchers’ declaration and management of conflicts of interest. Institutions are also expected to have a process for investigating allegations of unethical conduct by researchers, and for imposing consequences when unethical conduct is determined to have occurred.

A second focus of the institutional indicators is to ensure that institutions with their own research ethics committees adequately support those committees. Thus, one indicator asks whether such institutions ensure that their committees have access to necessary resources, from either the institution itself or external funders. Assessors are also asked to determine whether committees are provided with legal support, especially if an institutional research ethics committee has been challenged in court. Finally, assessors are asked to determine whether the institution facilitates the lodging of complaints by research participants or prospective participants about studies conducted by researchers affiliated to the institution, either through the institution itself or at a national or regional level. If a complaint system already exists for research participants at the national or regional level, the institution simply needs to include information about how to submit complaints through that system on the institution’s own website. If no such system exists, however, the institution would need to establish its own internal process for reviewing and responding to complaints.

## Implementation and next steps

The WHO research ethics oversight tool is not the first instrument designed to measure the quality of research ethics systems; a 2020 review found nine other such instruments in use by national regulatory agencies and private entities throughout the world.^18^ However, this tool is the first explicitly designed to be adaptable to all countries regardless of their existing level of research ethics capacity or available resources. Countries with limited resources and a low volume of research activity can use the tool to quickly establish baseline measurements through self-assessments that rely on simple data collection methods, such as reviews of paper records and discussions with research ethics committee staff. Countries with more developed systems might choose to implement externally conducted assessments involving more elaborate data collection methods, such as focus groups of research participants or regular retrospective reviews of research ethics committee meeting reports.

The WHO tool comes with a related user guide,^19^ which explains how the tool can be implemented through self-assessment, peer review or external evaluation. The tool and user guide have been translated into five languages, and plans are underway to develop an electronic data submission tool that will facilitate assessment and scoring. In addition, WHO has made extensive efforts to disseminate the tool and support its implementation, including sponsoring implementation workshops in the Lao People's Democratic Republic and Senegal in 2024; and in Lithuania, Rwanda and Tajikistan in 2025. In responding to demands for additional workshops, WHO is working with its partners, which include the Paul Ehrlich Institute; the Global Network of WHO Collaborating Centres for Bioethics; and regional networks such as the African Vaccine Regulatory Forum, the Forum for Ethical Review Committees in the Asian and Western Pacific Region, and the European research ethics committees' network. We hope that these workshops and other international collaborations will lead to the widespread adoption of the research ethics oversight tool in all WHO regions, and that this will stimulate lasting improvements in countries’ health research ecosystems.
